# PDCD5 Contributes to Airway Epithelial Cell Damage via Mitochondrial Pathway and Participates in COPD Pathogenesis

**DOI:** 10.1002/kjm2.70165

**Published:** 2025-12-25

**Authors:** Hu Shan, Rui Zhang, Yu‐er Li, Rui Li, Shao‐bo Ge, Jin Liu, Shi‐yuan Yao, Xia Yang, Tao Zhang, Ming Zhang

**Affiliations:** ^1^ Department of Respiratory and Critical Care Medicine The Second Affiliated Hospital of Xi'an Jiaotong University Xi'an Shaanxi China; ^2^ School of Pharmacy, Health Science Center Xi'an Jiaotong University Xi'an Shaanxi China

**Keywords:** airway epithelial cell, chronic obstructive pulmonary disease, cigarette smoke, mitochondria, PDCD5

## Abstract

Airway epithelial injury plays a critical role in the pathogenesis of chronic obstructive pulmonary disease (COPD). Mitochondrial dysfunction is implicated in this injury, while the underlying mechanism remains incompletely understood. RNA sequencing was conducted to identify key genes involved in mitochondrial dysfunction in airway epithelial injury induced by cigarette smoke extract (CSE). We identified 1981 significantly up‐regulated and 4952 down‐regulated differentially expressed genes (DEGs) in CSE‐treated airway epithelial cells. A protein–protein interaction network constructed from the DEGs revealed that several key genes were involved in CSE‐induced airway epithelial injury. Additionally, PDCD5 was identified as a hub gene potentially linked to mitochondrial dysfunction. PDCD5 expression was significantly increased in the airway epithelium of COPD patients and the corresponding experimental mice. The mRNA and protein expression levels of PDCD5 were significantly increased in concentration‐ and time‐dependent manners in airway epithelial cells treated with CSE. PDCD5 silencing significantly attenuated CSE‐induced mitochondrial reactive oxygen species (ROS) accumulation, mitochondrial membrane potential loss, and intracellular ATP depletion. Transmission electron microscopy revealed that PDCD5 siRNA treatment ameliorated CSE‐induced mitochondrial structural damage. Moreover, PDCD5 knockdown significantly reduced intracellular ROS accumulation, attenuated apoptosis increases, and inhibited cell viability decline in airway epithelial cells treated with CSE. Our findings demonstrate that PDCD5 contributes to airway epithelial cell damage through the mitochondrial pathway and participates in the pathogenesis of COPD, implicating it as a potential diagnostic biomarker and therapeutic target for COPD.

## 1 Introduction

Chronic obstructive pulmonary disease (COPD) is a significant and escalating global health problem, characterized by persistent respiratory symptoms and progressive airflow limitation [1]. It is a leading cause of morbidity and mortality worldwide, imposing a substantial socioeconomic burden [2]. Cigarette smoke exposure is the primary etiological factor, with other environmental pollutants and genetic predispositions also contributing to its pathogenesis [3]. The current therapeutic strategies primarily alleviate symptoms and reduce exacerbation rather than reverse COPD progression [4]. Thus, understanding the underlying molecular mechanisms is crucial for identifying new therapeutic targets.

As the primary barrier against inhaled noxious agents like cigarette smoke, injury to airway epithelial cells critically drives COPD initiation and progression [5]. Prolonged cigarette smoke exposure was reported to induce significant morphological and physiological impairments in airway epithelial cells [6]. This injury disrupts epithelial functions essential for mucociliary clearance, barrier integrity, and controlled mediator/protease release, triggering a self‐perpetuating cycle of chronic inflammation, oxidative stress, and tissue remodeling, and ultimately driving irreversible airflow limitation in COPD [7, 8]. Therefore, elucidating the mechanisms of airway epithelial injury is central to understanding the pathogenesis of COPD.

Mitochondria are master regulators of cellular energetics, redox homeostasis, calcium flux, and apoptosis, and their dysfunction contributes to airway epithelial injury in COPD [9]. Cigarette smoke extract (CSE) impairs mitochondrial respiration, increases reactive oxygen species (ROS) production, disrupts mitochondrial dynamics (fusion/fission), and triggers mitochondrial DNA damage [10, 11]. These alterations induce bioenergetic failure, exacerbate oxidative stress, activate inflammation, and promote airway epithelial cell death in the pathogenesis of COPD [12]. However, the molecular mechanisms underlying the contribution of mitochondrial dysfunction to airway epithelial injury in COPD remain incompletely understood.

Transcriptomic analysis, particularly RNA sequencing, enables the unbiased genome‐wide profiling of gene expression in complex diseases like COPD [13]. By analyzing the transcriptome of lung tissues or specific cell types (such as airway epithelial cells) from COPD patients, researchers have identified abnormally expressed genes involved in inflammation, oxidative stress response, extracellular matrix metabolism, immune cell activation, and cellular repair processes [14, 15]. RNA sequencing can provide valuable insights into COPD heterogeneity [16], potential biomarkers [17], and novel molecular mechanisms [18]. Therefore, our present study used RNA sequencing technology to explore the candidate genes in airway epithelial injury induced by CSE. Subsequently, critical roles of the hub gene in mitochondrial dysfunction and airway epithelial injury in COPD were investigated through in vivo and in vitro experiments.

## 2 Materials and Methods

### 2.1 Materials

Furongwang cigarettes (tar: 11 mg; nicotine: 1.2 mg; carbon monoxide: 11 mg) were purchased from China Tobacco Hunan Industry Co. Ltd. (Changsha, China). Lipopolysaccharide (LPS) was obtained from Sigma‐Aldrich (Category No. L2630, St. Louis, MO, USA), and the human airway epithelial cell line BEAS‐2B was acquired from Shanghai Cell Bank of Chinese Academy of Sciences (Shanghai, China). Programmed cell death 5 (PDCD5) siRNA was purchased from GenePharma Co. Ltd. (Suzhou, China). ELISA kits for interleukin‐6 (IL‐6) and tumor necrosis factor‐α (TNF‐α) were from Nanjing Jiancheng Bioengineering Institute (Jiangsu, China), and the PDCD5 ELISA kit was from Jingmei Biotechnology (Jiangsu, China). The kits used to measure intracellular ROS, cell apoptosis, mitochondrial membrane potential (MMP), and ATP were purchased from Beyotime Biotechnology (Shanghai, China). MitoSOX red dye was purchased from Thermo Fisher Scientific (Waltham, MA, USA), and cell counting kit‐8 (CCK‐8) was procured from Dojindo (Kumamoto, Japan). The antibodies for PDCD5, Bax, Bcl‐2, and GAPDH were obtained from ABclonal Technology (Wuhan, China). The enhanced chemiluminescence (ECL) kit was purchased from Thermo Fisher Scientific.

### 2.2 CSE Preparation

CSE was prepared by bubbling the smoke from one commercial cigarette into 10 mL of pre‐warmed RPMI 1640 medium using a vacuum pump as previously reported [19]. The pH of crude CSE was adjusted to 7.4, and it was filtered through a 0.22 μm filter. This solution was defined as 100% CSE, and the working concentration was prepared by dilution with culture medium.

### 2.3 RNA Sequencing

BEAS‐2B human airway epithelial cells were treated with 7.5% CSE for 24 h according to our previous experiments [20, 21], and total RNA was extracted using TRIzol reagent. RNA samples were sent to LC‐Bio Technology for high‐throughput sequencing on the Illumina platform (Hangzhou, China). Sequencing data were processed for quality control, alignment and quantification. Differentially expressed genes (DEGs) were identified using the DESeq2 package in R software (version 4.2.1), with thresholds of false discovery rate (FDR) < 0.05 and |log_2_ fold‐change (FC)| ≥ 2. The Gene Ontology (GO) enriched processes and Kyoto Encyclopedia of Genes and Genomes (KEGG) pathways of DEGs were analyzed using the online KOBAS tool (http://bioinfo.org/kobas). Protein–protein interaction (PPI) network was constructed using the Search Tool for the Retrieval of Interacting Genes/Proteins (STRING) and visualized in Cytoscape (version 3.9.1). The top 10 hub genes were identified using the maximal clique centrality method in the CytoHubba plugin.

### 2.4 Human Lung Tissues

A total of 11 subjects who underwent pulmonary lobectomy or segmentectomy for lung cancer were divided into control (*n* = 6) and COPD (*n* = 5) groups according to the diagnostic criteria for COPD. Lung tissue samples were collected at least 5 cm away from the tumor margin. The study protocol was approved by the Research Committee of Human Investigation of the Second Affiliated Hospital of Xi’an Jiaotong University (Approval number: 2024ER243), and informed consent was obtained from all participants.

### 2.5 Mouse Model of COPD

Male C57BL/6 mice (8 weeks old, 18–20 g) were purchased from the Animal Center of Xi'an Jiaotong University and randomly divided into the control (*n* = 8) and COPD model (*n* = 8) groups. The COPD mouse model was established by cigarette smoke exposure combined with intratracheal administration of LPS as described elsewhere with some modifications [22]. In brief, mice were administered LPS (1 μg/μL, 20 μL) intratracheally on days 1 and 14, and exposed to cigarette smoke on the other days for a total of 4 weeks. The mice were placed in sealed boxes and exposed to smoke from 10 commercial unfiltered cigarettes for 1 h, twice a day. Mice in the control group received tracheal infusion of saline (20 μL) and were exposed to room air using the same procedures. The animal study protocol was approved by the Biomedical Ethics Committee of Health Science Center of Xi'an Jiaotong University (Approval number: XJTUAE2025‐964).

On day 29, the mice were weighed and anesthetized by intraperitoneal injection of sodium pentobarbital (40 mg/kg). Peripheral blood samples were collected, followed by serum separation by centrifugation and storage at −80°C. Then 0.5 mL of sterile phosphate‐buffered saline (PBS) was instilled twice via tracheal cannula and recovered by gentle manual aspiration. The lavage fractions were centrifuged at 1500 rpm for 10 min at 4°C, and cell‐free supernatants were stored at −80°C for subsequent cytokine analysis. The cell pellet was resuspended in 1 mL of PBS, and then differential cell counts were calculated by Wright staining.

### 2.6 ELISA

IL‐6 and TNF‐α levels in bronchoalveolar lavage fluid (BALF), as well as serum PDCD5 levels in mice, were measured using the corresponding ELISA kits according to the manufacturers' instructions.

### 2.7 Pathological and Immunohistochemical Analyses

Human and mice lung tissues were fixed in 4% paraformaldehyde and embedded in paraffin. The paraffin‐embedded sections were stained with hematoxylin–eosin (HE) and observed under an optical microscope. Subsequently, immunohistochemical staining was performed on paraffin‐embedded lung tissue sections according to standard protocols, including deparaffinization, antigen retrieval, PDCD5 antibody incubation (1:200), diaminobenzidine development, and hematoxylin counterstaining. The protein expression of PDCD5 within airway epithelial cells was analyzed by Image J software.

### 2.8 PDCD5 siRNA Transfection

Airway epithelial cell BEAS‐2B was transfected with PDCD5 siRNA according to the manufacturer's protocol. The target sequences for PDCD5 were 5′‐CUGAUGAAGAUGACGAUUATT‐3′ (sense) and 5′‐UAAUCGUCAUCUUCAUCAGTT‐3′ (antisense). A nonspecific control siRNA was used as the negative control.

### 2.9 Real‐Time PCR

PDCD5 mRNA expression was determined by quantitative real‐time PCR. The forward primer sequence for PDCD5 was 5′‐CTTAGCCCAAGTTCTGGATCA‐3′, and the reverse primer sequence was 5′‐ACCTTGTTCTGATACCTTCTCAC‐3′. Results were expressed as fold changes relative to GAPDH levels using the 2^−∆∆CT^ method.

### 2.10 Western Blot

Following sodium dodecyl sulfate‐polyacrylamide gel electrophoresis separation, the protein was transferred onto PVDF membranes. The membranes were blocked with non‐fat milk and probed overnight at 4°C with specific antibodies against PDCD5 (1:1000), Bax (1:1000), Bcl‐2 (1:1000), and GAPDH (1:10000). Then, the membranes were incubated with a horseradish peroxidase‐conjugated secondary antibody and detected using an ECL kit. GAPDH served as the loading control for signal normalization.

### 2.11 Intracellular ROS Determination

Intracellular ROS is a general term for the total cellular burden of ROS originating from multiple organelles and determined using the fluorescent probe 2′,7′‐di‐chlorodihydrofluorescein diacetate (DCFH‐DA). Following treatments, airway epithelial cells were incubated with DCFH‐DA (10 μM) in PBS for 30 min at 37°C. The cells were then washed three times with PBS and imaged using inverted confocal microscopy (excitation 488 nm, emission 525 nm).

### 2.12 Apoptosis Assay

Cell apoptosis was detected with flow cytometry using the Annexin V‐FITC apoptosis detection kit. Briefly, airway epithelial cells were incubated with the FITC‐conjugated annexin V antibody and propidium iodide for 20 min at room temperature. Annexin V binding was analyzed by FACScan. The apoptosis rate was expressed as the percentage of annexin V‐FITC‐positive cells to the total number of cells.

### 2.13 Detection of Mitochondrial ROS

Mitochondrial ROS is a major ROS subset specifically generated within mitochondria and measured using the MitoSOX red, a live‐cell permeant dye that selectively targets mitochondria. Airway epithelial cells were incubated with 5 μM MitoSOX Red for 10 min at 37°C. After washing with PBS, fluorescence intensity was determined by flow cytometry at excitation and emission wavelengths of 510 nm and 580 nm, respectively.

### 2.14 MMP Measurement

Airway epithelial cells were incubated with JC‐1 staining solution at 37°C for 20 min and then washed three times with JC‐1 staining buffer. Fluorescent cells were observed by confocal microscopy. Bright red fluorescence represents the J‐aggregate, and green fluorescence represents the J‐monomer. MMP was quantified by calculating the ratio of red to green fluorescence intensities.

### 2.15 Transmission Electron Microscopy

Airway epithelial cells were fixed with 2.5% glutaraldehyde and 1% osmium tetroxide, followed by dehydration in a graded ethanol series. Ultrathin sections were mounted on 400‐mesh copper grids, stained with uranyl acetate and lead citrate, and then observed under a transmission electron microscope.

### 2.16 Intracellular ATP Measurement

Intracellular ATP levels were quantified using a firefly luciferase‐based ATP assay kit according to the manufacturer's instructions, and luminescence was detected using a fluorescence microplate reader.

### 2.17 Cell Viability Assay

Airway epithelial cells were incubated with CCK‐8 solution at 37°C for 2 h, and the absorbance was determined at 450 nm by an automated microplate reader.

### 2.18 Statistical Analysis

All data are presented as mean ± standard deviation (SD) and analyzed using SPSS 26.0 software. Student's *t*‐test and one‐way analysis of variance test (ANOVA) were used for inter‐group comparisons. A *p‐*value of less than 0.05 was considered significant.

## 3 Results

### 3.1 Transcriptomic Alterations in Airway Epithelial Cells Treated With CSE

**FIGURE 1:**
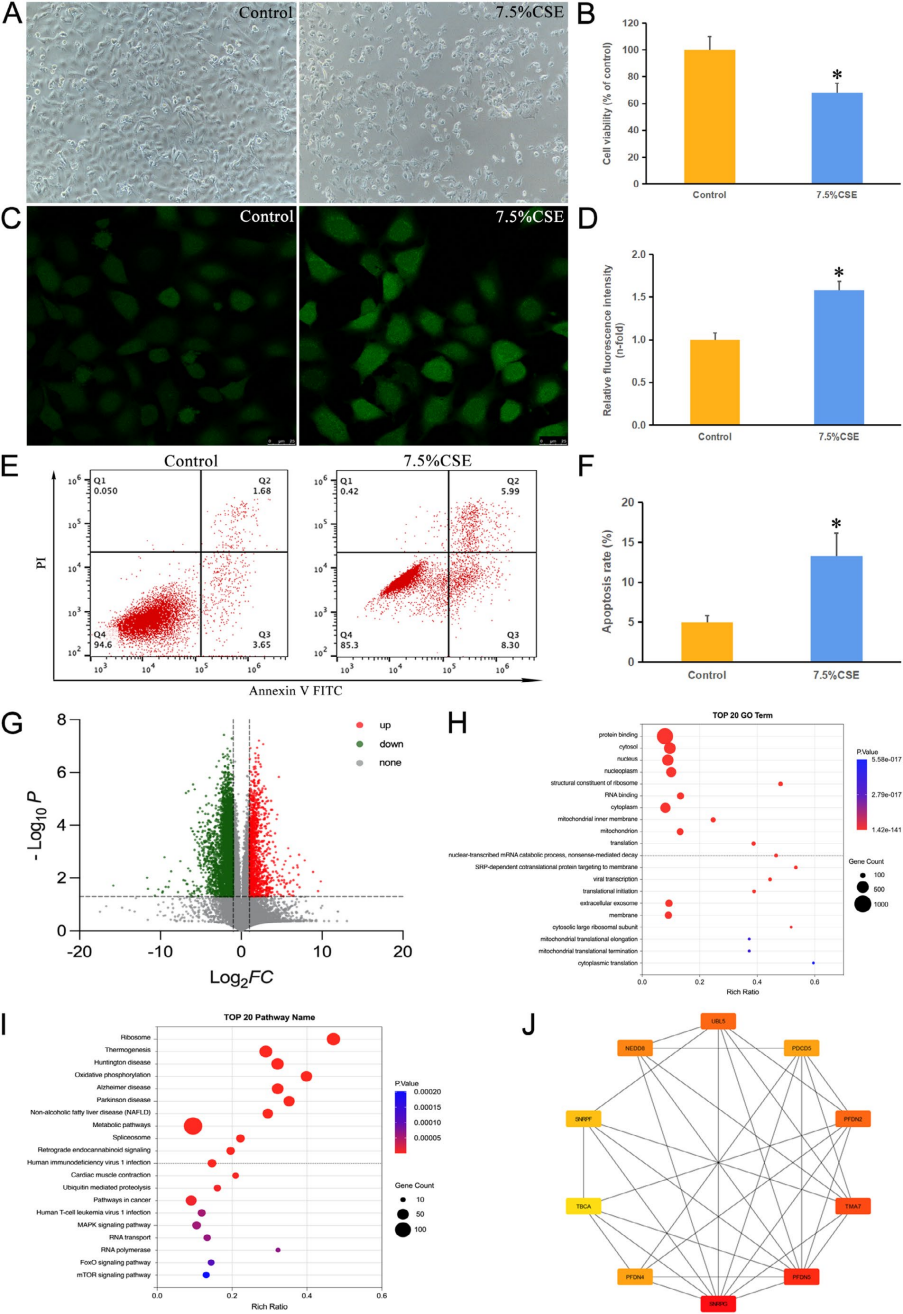
Transcriptomic alterations in airway epithelial cells treated with CSE. The morphology of airway epithelial cells was observed under light microscopy (×200) (A). Cell viability was measured by the CCK‐8 assay (B). Intracellular reactive oxygen species (ROS) levels were detected by confocal microscopy (C), and quantitative analysis revealed a significant increase in ROS levels in airway epithelial cells treated with 7.5% CSE (D). Cell apoptosis was assessed by flow cytometry (E), showing a significantly elevated apoptosis rate in airway epithelial cells exposed to 7.5% CSE (F). RNA sequencing was performed on airway epithelial cells treated with 7.5% CSE, and the volcano plot (G), GO enrichment analysis (H), KEGG pathway analysis (I), and PPI network (J) were generated. Data are expressed as mean ± SD from three independent experiments. *p < 0.05 compared to the control group.

Following treatment with 7.5% CSE for 24 h, light microscopy revealed obvious morphological changes in airway epithelial cells (Figure 1A). Control cells exhibited intact morphology, dense packing, and firm attachment. In contrast, cells treated with 7.5% CSE appeared shrunken and irregularly shaped, accompanied by a marked reduction in density. The CCK‐8 assay demonstrated significantly decreased cell viability in the 7.5% CSE‐treated group compared with the control group (*p* < 0.01, Figure 1B). Intracellular ROS levels in the airway epithelial cells treated with 7.5% CSE were significantly higher than those in control cells (*p* < 0.01, Figure 1C,D). Moreover, the apoptosis rate of airway epithelial cells was significantly increased to approximately 13.3% following the treatment with 7.5% CSE for 24 h (*p* < 0.01, Figure 1E,F).

We performed RNA sequencing on airway epithelial cells treated with 7.5% CSE to identify key molecules involved in airway epithelial damage during COPD pathogenesis. The volcano plot revealed 1981 significantly up‐regulated and 4952 down‐regulated DEGs (Figure 1G). GO enrichment analysis demonstrated that DEGs were mainly focused on several biological terms, including protein binding, cytosol, nucleus, nucleoplasm, structural constituent of ribosome, RNA binding, cytoplasm, mitochondrial inner membrane, and mitochondrion (Figure 1H). KEGG pathway analysis showed that oxidative phosphorylation was one of the top‐enriched pathways for DEGs, along with other mitochondria‐associated pathways (Figure 1I). Further PPI network constructed from the DEGs revealed several key hub genes involved in CSE‐induced airway epithelial injury, including *SNRPG*, *PFDN2*, *PFDN4*, *PFDN5*, *TMA7*, *PDCD5*, *UBL5*, *NEDD8*, *SNRPF*, and *TBCA* (Figure 1J). Among these 10 key genes, PDCD5 has been reported to be potentially associated with mitochondrial function [23].

### 3.2 Protein Expression of PDCD5 in the Airway Epithelium of COPD

**FIGURE 2:**
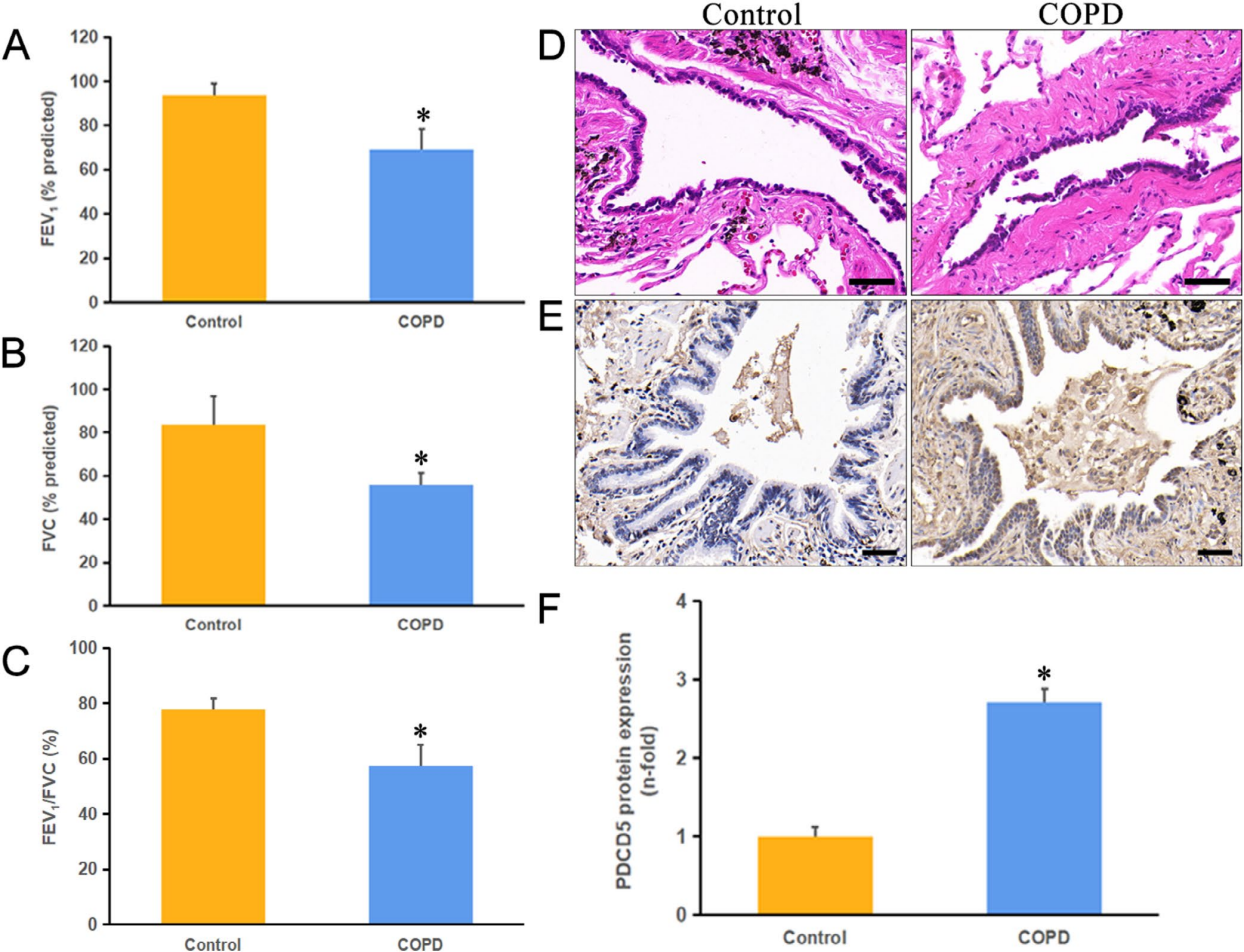
Protein expression of PDCD5 in the airway epithelium of control subjects and COPD patients. Forced expiratory volume in 1 s (FEV_1_) (A), forced vital capacity (FVC) (B), and FEV_1_/FVC ratio (C) were significantly decreased in COPD patients compared with the controls. Representative HE staining of lung tissue sections from control subjects and COPD patients (D). Representative immunohistochemical images of PDCD5 in human airway epithelium (E), with quantification showing significantly increased PDCD5 protein expression in COPD patients (F). The scale bar represents 50 μm. Data are shown as mean ± SD (*n* = 6 and 5, respectively). *p < 0.05 compared to the control group.

Compared with the controls, forced expiratory volume in 1 s (FEV_1_), forced vital capacity (FVC), and FEV_1_/FVC of COPD patients were all significantly decreased (69.12% ± 9.08% vs. 93.89% ± 4.89%, 55.66% ± 5.59% vs. 83.70% ± 13.09%, and 57.37% ± 7.53% vs. 77.42% ± 5.15%, respectively, *p* < 0.01, Figure 2A–C). The control group exhibited normal small airway and alveolar structures, whereas COPD patients showed thickened bronchial walls, narrowed small airways, partially discontinuous alveolar septa, and dilated alveolar spaces (Figure 2D). PDCD5 protein expression in the airway epithelium was determined by immunohistochemical analysis. The results showed that its expression (brown staining) in airway epithelial cells was significantly increased in COPD patients compared to controls (*p* < 0.01, Figure 2E,F).

**FIGURE 3:**
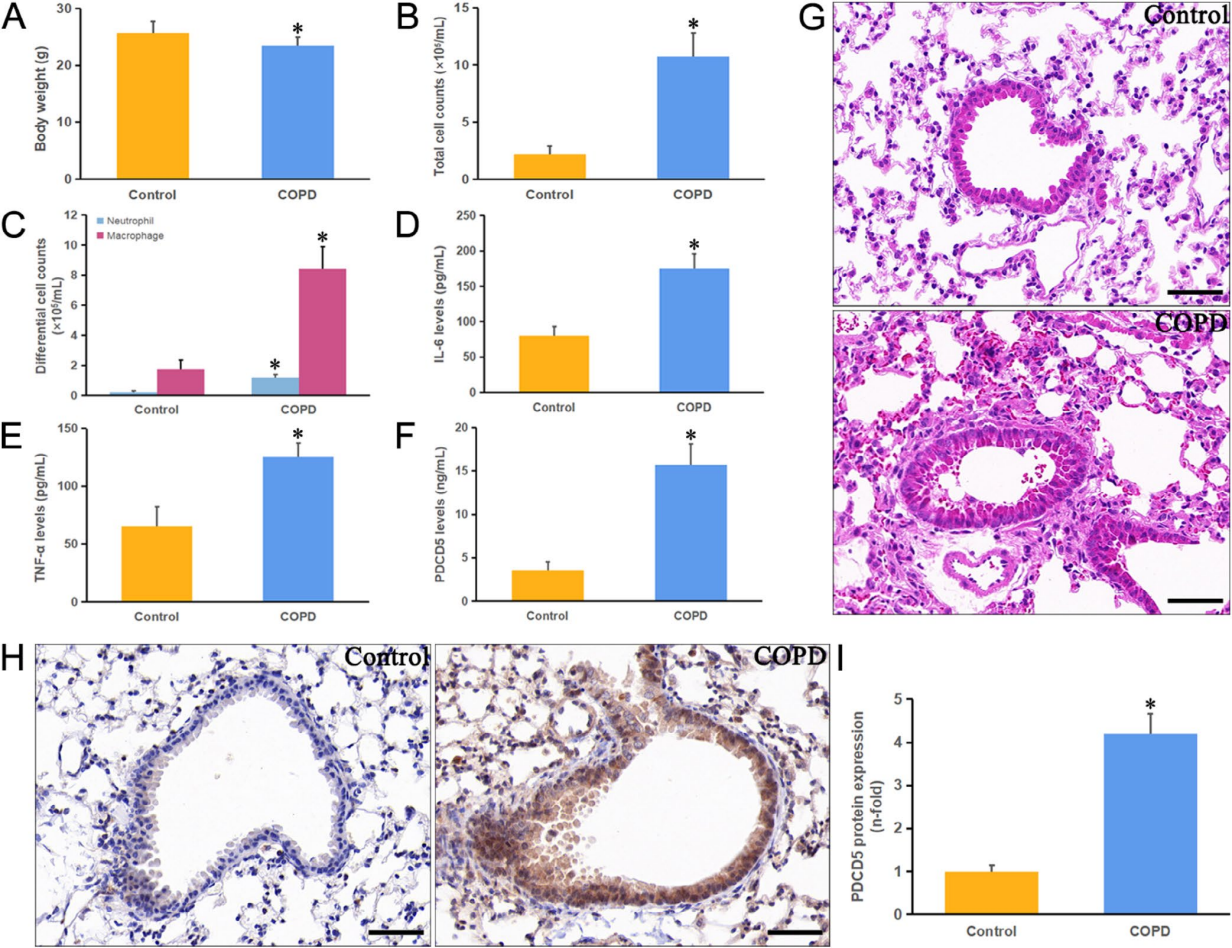
Protein expression of PDCD5 increased in the airway epithelium of COPD mice. The body weight of COPD mice was significantly decreased compared to controls (A). The numbers of total cells, macrophages, and neutrophils in bronchoalveolar lavage fluid (BALF) from COPD mice were significantly increased (B, C). Levels of IL‐6 and TNF‐α in BALF (D, E), and PDCD5 in serum (F) were significantly higher in COPD mice than in controls. Representative HE staining of lung tissue sections from control and COPD mice (G). PDCD5 expression in mouse airway epithelium was determined by immunohistochemistry (H), showing significantly increased protein expression in COPD mice (I). The scale bar represents 50 μm. Data are shown as mean ± SD (*n* = 8 for each group). *p < 0.05 compared to the control group.

The body weight of COPD mice was lower than that of the control group (23.46 ± 1.49 g vs. 25.69 ± 2.00 g, *p* < 0.05, Figure 3A). The total number of cells in BALF was significantly increased in COPD mice compared to control mice, with values of (10.72 ± 2.10) × 10^5^/L vs. (2.20 ± 0.73) × 10^5^/L (*p* < 0.01, Figure 3B). Further differential cell counts showed that the numbers of macrophages and neutrophils in BALF from COPD mice were both significantly higher than those in the control mice (*p* < 0.01, Figure 3C). In addition, the concentration of IL‐6 and TNF‐α in BALF and PDCD5 in serum were significantly increased in the COPD group compared with the control group (*p* < 0.01, Figure 3D–F). Mouse lung tissue sections were observed under a light microscope, and representative images are shown in Figure 3G. Bronchi and alveolar structures were well‐defined in the control group, with no notable evidence of mucosal destruction or inflammatory cell infiltration. In contrast, COPD mice exhibited disorganized lung architecture with significant destruction, characterized by ruptured alveolar walls, dilated alveolar spaces, and prominent inflammatory cell infiltration around the bronchi. Immunohistochemical analysis demonstrated that PDCD5 protein expression in the airway epithelium of COPD mice was significantly increased compared with the control mice (*p* < 0.01, Figure 3H,I).

### 3.3 The Role of PDCD5 in CSE‐Induced Airway Epithelial Injury

**FIGURE 4:**
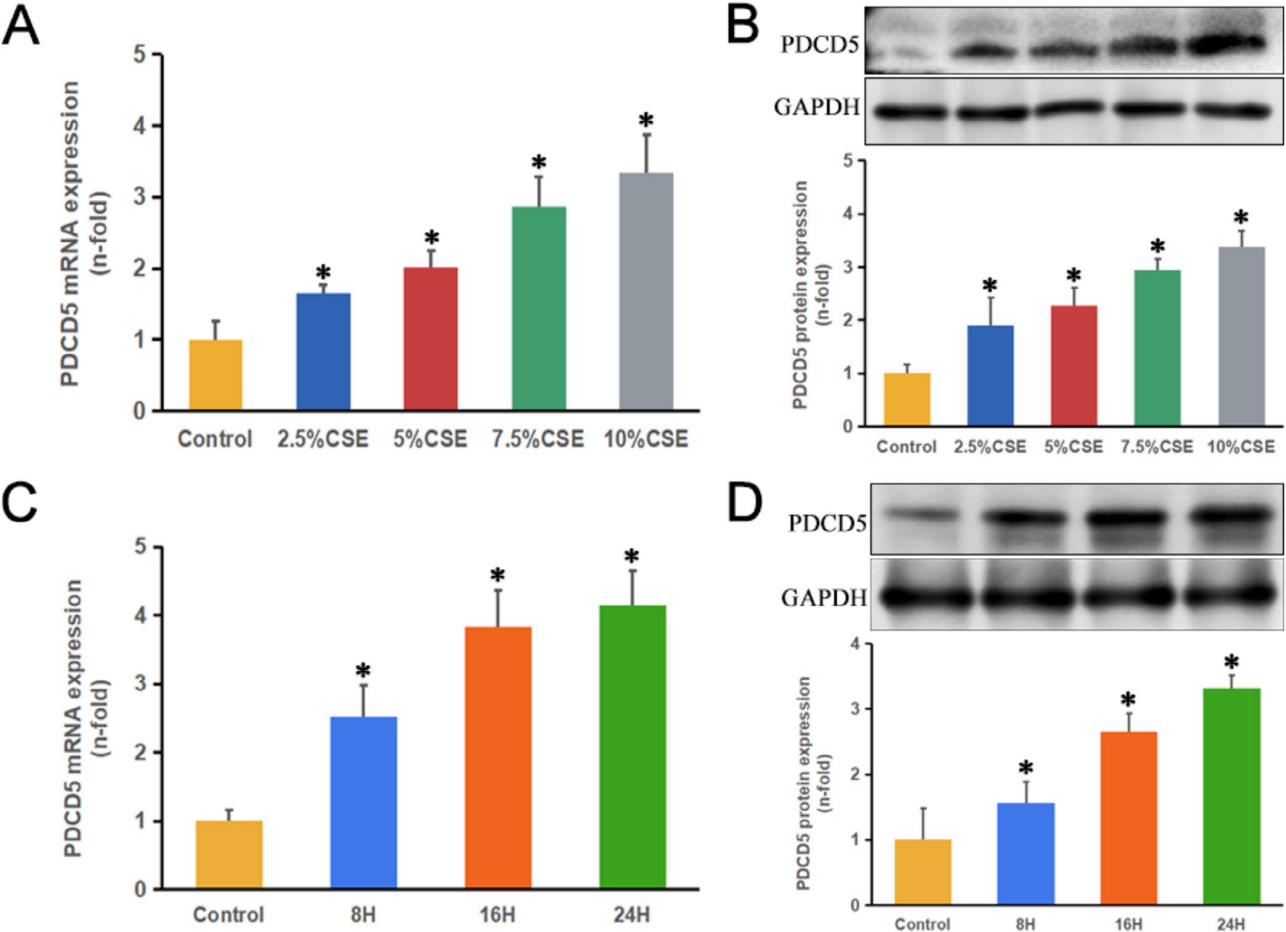
PDCD5 expression was increased in airway epithelial cells treated with CSE. Stimulation of airway epithelial cells with CSE for 24 h significantly increased PDCD5 expression in a concentration‐dependent manner, as measured by real‐time PCR (A) and western blot (B). The mRNA (C) and protein (D) expression levels of PDCD5 were significantly increased in a time‐dependent manner when airway epithelial cells were stimulated with 7.5% CSE. All statistical data were obtained from three independent experiments and presented as mean ± SD. *p < 0.05 compared to the control group.

After CSE treatments with various concentrations (2.5%, 5%, 7.5%, and 10%) or different time intervals (8, 16, and 24 h), the alterations of PDCD5 expression were determined by western blot and real‐time PCR. The results showed that PDCD5 mRNA and protein expression levels were significantly increased in a concentration‐dependent manner in airway epithelial cells treated with 2.5%–10% CSE for 24 h (*p* < 0.05, Figure 4A,B). Additionally, 7.5% CSE increased PDCD5 mRNA and protein expression in a time‐dependent manner in airway epithelial cells as shown in Figure 4C,D (*p* < 0.05).

**FIGURE 5:**
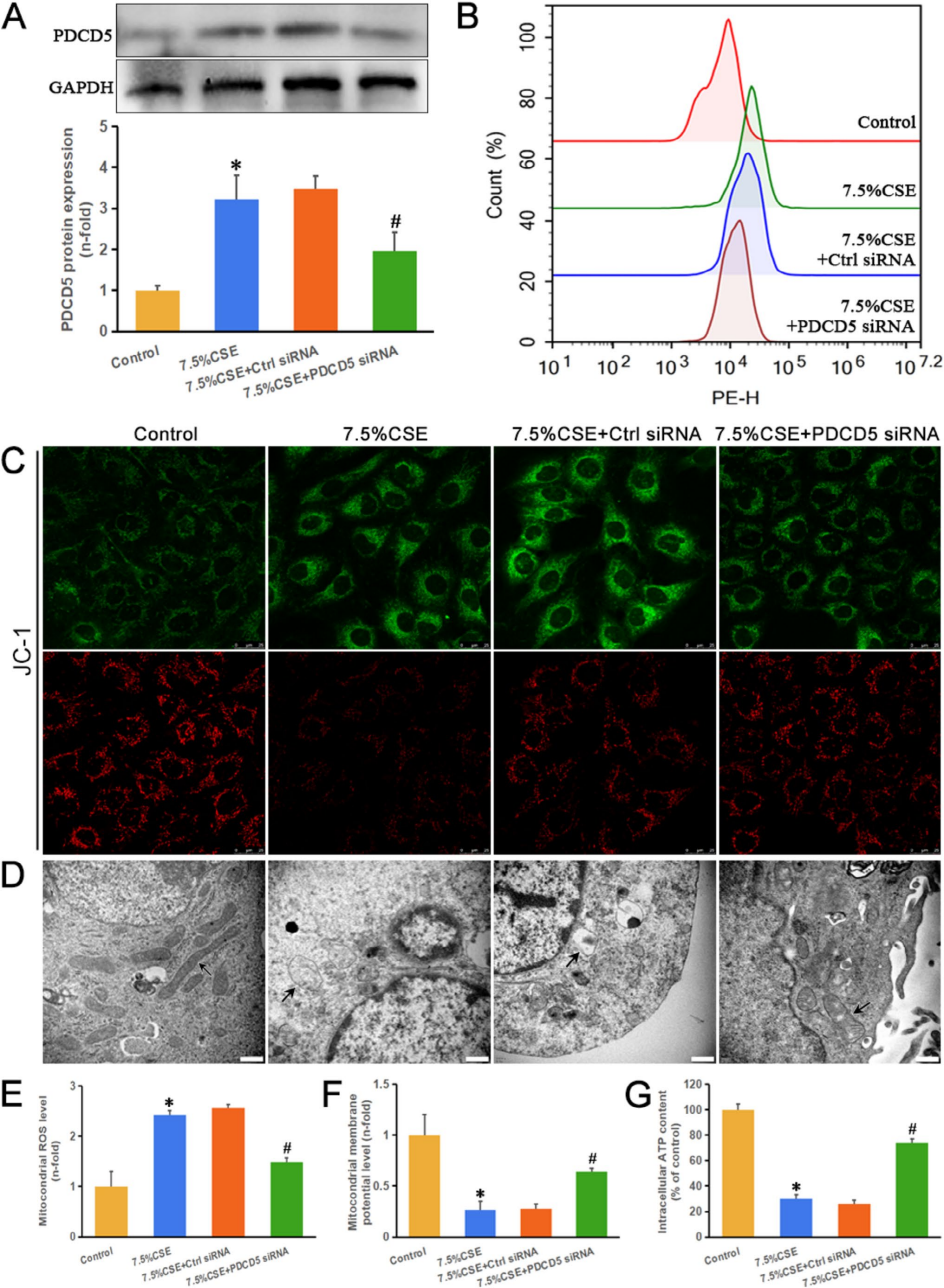
PDCD5 silencing attenuated airway epithelial mitochondrial injury induced by CSE. PDCD5 protein expression was significantly down‐regulated in airway epithelial cells treated with 7.5% CSE and PDCD5 siRNA (A). Mitochondrial reactive oxygen species (ROS) content in airway epithelial cells was measured by flow cytometry (B). Mitochondrial membrane potential was assessed by confocal microscopy with representative images shown (C). Transmission electron microscopy revealed that PDCD5 silencing significantly improved mitochondrial structural damage of airway epithelial cells induced by 7.5% CSE. The arrows indicate mitochondrial alterations, and the scale bar represents 500 nm (D). Quantitative analysis showed that PDCD5 silencing significantly attenuated 7.5% CSE‐induced mitochondrial ROS accumulation (E), mitochondrial membrane potential loss (F), and intracellular ATP depletion (G). Results are expressed as mean ± SD from three independent experiments. *p < 0.05 versus control group, and #*p* < 0.05 versus 7.5% CSE group.

PDCD5 protein expression was significantly down‐regulated in airway epithelial cells transfected with PDCD5 siRNA and exposed to 7.5% CSE (*p* < 0.05, Figure 5A). Flow cytometry analysis indicated that 7.5% CSE significantly increased mitochondrial ROS levels in airway epithelial cells. This increase was significantly attenuated when airway epithelial cells were transfected with PDCD5 siRNA (*p* < 0.05, Figure 5B,E). MMP levels were significantly decreased in 7.5% CSE‐treated cells compared with the control cells, while this decline was significantly prevented by pretreatment with PDCD5 siRNA (*p* < 0.05, Figure 5C,F). Transmission electron microscopy showed profound mitochondrial structural damage in 7.5% CSE‐treated airway epithelial cells, including swollen cristae and vacuolization, which were ameliorated by PDCD5 siRNA pretreatment (Figure 5D). Moreover, PDCD5 silencing significantly inhibited the decline of ATP levels in airway epithelial cells treated with 7.5% CSE (*p* < 0.05, Figure 5G).

**FIGURE 6:**
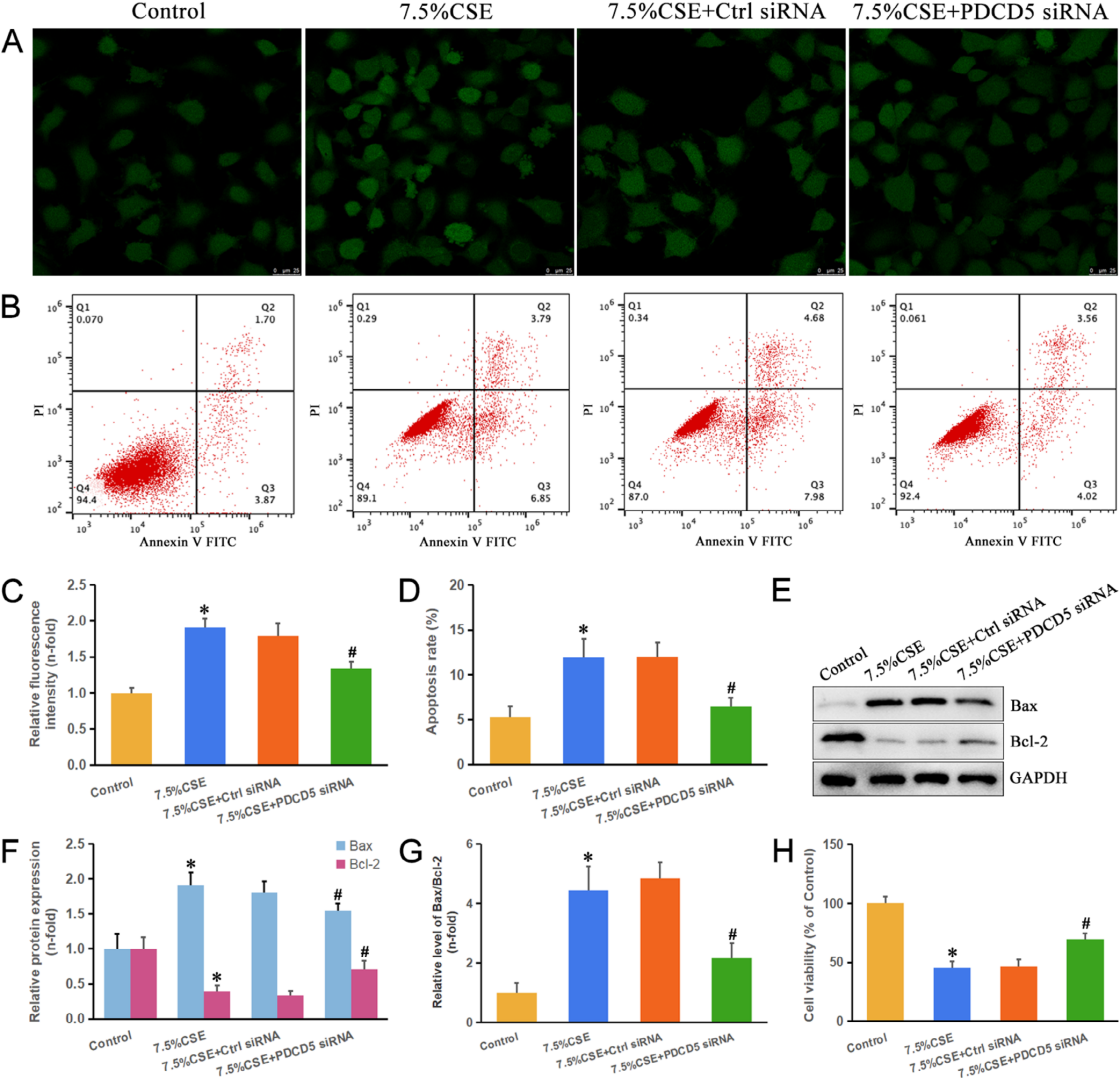
PDCD5 knockdown alleviated airway epithelial injury induced by CSE. Intracellular reactive oxygen species (ROS) levels were detected by confocal microscopy (A), and cell apoptosis was assessed by flow cytometry (B). Quantitative analysis showed that PDCD5 knockdown significantly reduced intracellular ROS accumulation and attenuated apoptosis increases in airway epithelial cells treated with 7.5% CSE (C, D). Protein expression of Bax and Bcl‐2 was determined by western blot, and representative blots are presented (E). PDCD5 siRNA significantly reversed the up‐regulation of Bax, down‐regulation of Bcl‐2, and the increased Bax/Bcl‐2 ratio in airway epithelial cells treated with 7.5% CSE (F, G). Treatment with PDCD5 siRNA also significantly attenuated CSE‐induced decline in cell viability (H). All the data are shown as mean ± SD from three independent experiments. *p < 0.05 versus control group, and #*p* < 0.05 versus 7.5% CSE group.

Intracellular ROS levels, apoptosis degree, and cell viability were determined in airway epithelial cells treated with PDCD5 siRNA and 7.5% CSE to further elucidate the potential role of PDCD5 in airway epithelial injury in COPD. PDCD5 silencing significantly reduced intracellular ROS levels and apoptosis rate in 7.5% CSE‐treated airway epithelial cells (*p* < 0.05, Figure 6A–D). Additionally, 7.5% CSE significantly up‐regulated Bax expression, down‐regulated Bcl‐2 expression, and increased the Bax/Bcl‐2 ratio in airway epithelial cells (*p* < 0.01). Moreover, these alterations were significantly prevented by treatment with PDCD5 siRNA (*p* < 0.05, Figure 6E–G). Cell viability in the 7.5% CSE‐treated group was significantly decreased compared with the control group (*p* < 0.01), and this decrease was significantly inhibited by PDCD5 siRNA treatment (*p* < 0.05, Figure 6H).

## 4 Discussion

COPD is primarily triggered by the chronic inhalation of noxious particles, especially cigarette smoke, and involves inflammation, oxidative stress, as well as protease‐antiprotease imbalance [24]. Airway epithelial cell injury is the initial event during COPD progression, and its mitochondrial dysfunction plays a crucial role in this process [9]. It has been reported that CSE induced mitochondrial dysfunction by upregulating aryl hydrocarbon receptor repressor expression, which potentially promotes unprogrammed/immunogenic airway epithelial cell death [25]. Our previous study has also demonstrated that cyclophilin D (a component of mitochondrial permeability transition pore) contributed to airway epithelial injury by regulating mitochondrial function in the pathogenesis of COPD [21]. Therefore, investigating the airway epithelial cell injury process from a mitochondrial perspective facilitates the elucidation of COPD pathogenesis.

Transcriptomic analysis was performed on an in vitro airway epithelial cell model of COPD to systematically identify novel genes involved in mitochondria‐associated airway epithelial injury in COPD [21, 26]. RNA sequencing and PPI network analysis showed that the top 10 key genes in CSE‐treated airway epithelial cells were *SNRPG*, *PFDN2*, *PFDN4*, *PFDN5*, *TMA7*, *PDCD5*, *UBL5*, *NEDD8*, *SNRPF*, and *TBCA*. Among these genes, PDCD5 emerged as a hub candidate gene for further investigation due to its significant magnitude of change and potential mitochondrial relevance [23]. PDCD5, originally identified as an apoptosis‐accelerating protein, is widely expressed and highly conserved across species [23]. This protein displays pleiotropic function, with roles in programmed cell death and immune regulation [27]. It also accelerates apoptosis in multiple cell types upon stimulation [27].

Accumulating data have demonstrated that PDCD5 is involved in the pathogenesis of tumors [28], autoimmune diseases [29], and inflammatory processes [30]. The levels of PDCD5 in serum and synovial fluid in patients with rheumatoid arthritis were significantly higher than those in the patients with osteoarthritis and healthy controls [31]. It has also been reported that PDCD5 expression was significantly elevated in ovalbumin‐induced allergic asthma, which was significantly correlated with inflammatory cell numbers in BALF and lung function [32]. PDCD5 expression was significantly increased in the lung tissues of patients with idiopathic pulmonary fibrosis and its mouse model, and pulmonary fibrosis was significantly diminished by club‐cell‐specific deletion of the PDCD5 gene [33]. However, the role of PDCD5 in COPD pathogenesis is still unknown. Our present study demonstrated that serum PDCD5 levels were significantly increased in COPD mice, and PDCD5 protein expression was elevated in the airway epithelium of both COPD patients and the mouse model. Further in vitro studies demonstrated that the mRNA and protein expression levels of PDCD5 were significantly increased in concentration and time‐dependent manners in airway epithelial cells treated with CSE. This consistent elevation in human disease, a relevant mouse model, and CSE‐treated epithelial cells strongly suggests that PDCD5 plays an important role in airway epithelial injury in the pathogenesis of COPD.

To illustrate the role of PDCD5 in airway epithelial damage in COPD, a series of experiments was carried out. Consistent with previous reports [34, 35], our present study has also demonstrated that CSE could induce notable mitochondrial structure and function damage in airway epithelial cells. This injury was significantly improved by pretreatment with PDCD5 siRNA, indicating that PDCD5 may induce airway epithelial mitochondrial damage in the pathogenesis of COPD. Furthermore, the specific reduction in early apoptosis upon PDCD5 silencing is a key finding supporting its pivotal role in the mitochondrial apoptotic pathway [36]. As PDCD5‐mediated MMP loss is an early apoptotic event, this explains why PDCD5 knockdown attenuated CSE‐induced early apoptosis in the airway epithelial cells. The unaltered late apoptotic population further corroborates that PDCD5 functions upstream in the apoptotic cascade, prior to the execution phases involving DNA fragmentation and apoptotic body formation. In addition, pretreatment with PDCD5 siRNA significantly improved airway epithelial cell damage, as evidenced by reduced intracellular ROS levels and apoptosis degree, as well as elevated cell viability. These findings indicate that PDCD5 silencing may ameliorate airway epithelial cell injury in the progression of COPD. However, whether PDCD5 up‐regulation in the airway epithelium is a causative factor in COPD remains unclear. This represents a limitation of our study, and more experiments are needed to establish a direct causal role of PDCD5 in COPD pathogenesis, such as in vivo inhibition or knockdown of PDCD5.

In conclusion, our study has demonstrated that PDCD5 promoted airway epithelial cell injury through the mitochondrial pathway, suggesting its critical role in COPD pathogenesis. These findings not only elucidate a novel mitochondria‐related mechanism in COPD pathogenesis but also provide a potential diagnostic biomarker and therapeutic target for COPD.

## Funding

This study was supported by Key R&D Program of Health Scientific Research Innovation Capacity Enhancement Plan of Shaanxi Province (No. 2025YF‐11), IIT Clinical Research Fund of the Second Affiliated Hospital of Xi'an Jiaotong University (No. IIT031), and Xi'an Jiaotong University Medical Development Fund (No. XJYG2025‐SFJJ004).

## Conflicts of Interest

The authors declare no conflicts of interest.

## Data Availability

The data that support the findings of this study are available from the corresponding author upon reasonable request.
